# Alteration of coupling between brain and heart induced by sedation with propofol and midazolam

**DOI:** 10.1371/journal.pone.0219238

**Published:** 2019-07-17

**Authors:** Dong-Ok Won, Bo-Ram Lee, Kwang-Suk Seo, Hyun Jeong Kim, Seong-Whan Lee

**Affiliations:** 1 Department of Brain and Cognitive Engineering, Korea University, Seoul 02841, Republic of Korea; 2 Department of Artificial Intelligence, Korea University, Seoul 02841, Republic of Korea; 3 Department of Dental Anesthesiology, Seoul National University Dental Hospital, Seoul 03080, Republic of Korea; Imperial College London, UNITED KINGDOM

## Abstract

For a comprehensive understanding of the nervous system, several previous studies have examined the network connections between the brain and the heart in diverse conditions. In this study, we identified coupling between the brain and the heart along the continuum of sedation levels, but not in discrete sedation levels (*e*. *g*., wakefulness, conscious sedation, and deep sedation). To identify coupling between the brain and the heart during sedation, we induced several depths of sedation using patient-controlled sedation with propofol and midazolam. We performed electroencephalogram (EEG) spectral analysis and extracted the instantaneous heart rate (HR) from the electrocardiogram (ECG). EEG spectral power dynamics and mean HR were compared along the continuum of sedation levels. We found that EEG sigma power was the parameter most sensitive to changes in the sedation level and was correlated with the mean HR under the effect of sedative agents. Moreover, we calculated the Granger causality (GC) value to quantify brain-heart coupling at each sedation level. Additionally, the GC analysis revealed noticeably different strengths and directions of causality among different sedation levels. In all the sedation levels, GC values from the brain to the heart (GC_b→h_) were higher than GC values from the heart to the brain (GC_h→b_). Moreover, the mean GC_b→h_ increased as the sedation became deeper, resulting in higher GC_b→h_ values in deep sedation (1.97 ± 0.18 in propofol, 2.02 ± 0.15 in midazolam) than in pre-sedation (1.71 ± 0.13 in propofol, 1.75 ± 0.11 in midazolam; *p* < 0.001). These results show that coupling between brain and heart activities becomes stronger as sedation becomes deeper, and that this coupling is more attributable to the brain-heart direction than to the heart-brain direction. These findings provide a better understanding of the relationship between the brain and the heart under specific conditions, namely, different sedation states.

## Introduction

Various internal organs form a complex network, and this physiological network regulates body balance by maintaining homeostasis and adapting to internal and external stimulations [1]. Among these organs, the brain is the main organ in the central nervous system and the heart is the main organ affected by the autonomic nervous system. These organs, which are representative for or affected by the nervous systems, have been studied in the context of interaction networks as well as in terms of the activity of individual organs. Among the diverse neural mechanisms involved in the brain-heart network, the central autonomic network (CAN), which is an internal regulation system through which the brain controls visceromotor, neuroendocrine, and behavioral responses in diverse environments, has been considered as the main route for the connection between the brain and the heart. The route from the frontal cortex to the sinoatrial node of the heart has been continuously investigated as the main CAN pathway of the information flow [2, 3]. This information flow between the brain and the heart through CAN has been demonstrated in neuroanatomical studies performed in various physiological conditions (*e*. *g*., emotion alteration, resting states, sleep level alteration, and immune system alteration) [4–10]. Recently, brain-heart coupling analysis based on CAN was performed at different levels of consciousness [11–18]. In particular, alteration of the consciousness level by anesthesia was studied to analyze brain-heart coupling. The information flow in the brain-heart network works in both directions. The heart to brain information flow transmits visceral information and has been demonstrated using heartbeat-evoked potential (HEP) pathways via visceral cardiac afferents to the frontal cortical area [19–22].

Anesthesia-induced altered levels of consciousness have been used in clinical practice for medical and surgical procedures [23]. In this context, consciousness levels have been distinguished using brain or heart activities. Initially, attempts to distinguish consciousness levels used heart activity parameters such as heart rate (HR) and heart rate variability (HRV), given that heart activity changes over different consciousness levels [11, 24]. However, using the heart activity has critical limitations because its changes are indirect measures for discriminating consciousness levels. Instead, electroencephalography (EEG), which measures the brain’s electrical activity [25–28], has been proposed as a more direct measure of consciousness levels [29–31].

Recently, there has been an increase in adopting various approaches to discern the consciousness levels using brain-heart interaction as well as individual activities of each organ [14]. Most studies focused on differentiating stable states of consciousness without considering the gradual change of consciousness states [14, 32]. Since the state of consciousness changes continuously and gradually and each consciousness level is only vaguely defined in the clinical field (*e*. *g*., purposeful response to auditory or tactile stimulation defined as moderate sedation and purposeful response after repeated or painful stimulation defined as deep sedation [33]), the dynamics of consciousness levels need to be investigated on a continuum of sedation levels and analyzed on the stationary sedation level at the same time.

In this study, we investigated the coupling between the cortical and cardiac activities using EEG and ECG, respectively, together with the dynamic continuum of sedation levels. Different sedation levels from wakefulness to deep sedation were repeatedly induced for the analysis of the dynamics of sedation level on a continuum using propofol and midazolam. We induced this dynamically changing sedation levels using patient-controlled sedation (PCS), which facilitated the induction and maintenance of more obvious dynamics of sedation levels. This approach also allowed us to investigate time-varying coupling analysis between the brain and the heart. The natural consciousness alteration process is a continuous change and not a discrete change. In our study, we adopted this continuously changing process of consciousness and attempted to reveal the coupling pattern between the brain and the heart in this real consciousness changing process. We extracted and used EEG spectral sigma power and mean HR as representative physiological signals for brain and heart activity, respectively. Observing the time-varying dynamics of these two representative signals, we also identified coupling between the sigma power and mean HR along the dynamics of sedation levels on a continuum. Moreover, Granger causality (GC) values were calculated between the sigma power and mean HR to investigate the coupling between the two signals across the different sedation levels. We revealed that there was different causal link between sigma power and mean HR along the continuum of sedation levels. In both the propofol and midazolam groups, the mean of GC value increased as the depth of sedation level deepened, regardless of the direction of causal links (GC_b→h_ and GC_h→b_). Even though we did not expect significant differences for GC_h→b_ among the sedation levels, GC_h→b_ showed a similar trend with GC_b→h_.

In our study, we characterized the coupling pattern between different physiological activities during sedation and provided a more comprehensive understanding of brain-heart coupling under the action of sedative agents. Our findings may be applicable in the clinical field to monitor sedation levels.

## Materials and methods

### Participants

This study was approved by the institutional review board (IRB) at Seoul National University Dental Hospital (CEM15002). In addition, this study was registered (registration number KCT0001618) with the clinical research information service (CRiS), Republic of Korea (URL: https://cris.nih.go.kr/cris/index.jsp). All experiments were performed under monitoring by an anesthesiologist. A total of 60 participants (45 male and 15 female participants) were enrolled in this study (mean age ± standard deviation (SD) 26.87 ± 5.37 years) and fulfilled the American Society of Anesthesiologists (ASA) class I. Written informed consent was obtained from all participants. We allocated ten participants to each group with a total of six groups (two types of anesthetics and three doses). 20 participants in the group of low-dose propofol and midazolam were excluded from further analysis because they did not reach deep sedation or unconsciousness. In additional, we analyzed a total of 35 out of the 40 participants of these two groups (propofol middle-dose and high-dose groups: n = 9 each; midazolam middle-dose and high-dose groups: n = 8 and 9, respectively). The five remaining participants were excluded due to insufficient artifact-free EEG data caused by violent movements (*i*. *e*., frequent head movements) and lack of data on consciousness change throughout the experiment. Table 1 summarizes the demographic information of the participants that were included in the analysis.

**Table 1:** Demographic information for the study.

Study demographics (N = 35)				
	Mean±std.			Number
**Age**	27.4±6.37	**Sex**	Male	26
**Weight(kg)**	69.03±12.25		Female	9
**Height(cm)**	172.25±8.14	**Handedness**	right	33
**BMI(kg/m^2^)**	23.11±2.84		left	1
			both	1

### Experimental setup

The EEG signals were measured by the BrainAmp amplifier and 62 Ag/AgCl EEG electrodes (Brain Products GmbH, Germany). ECG, blood pressure (BP), end-tidal carbon-dioxide (EtCO_2_), and peripheral oxygen saturation (SpO_2_) were non-invasively and continuously monitored by a standard patient monitoring system (BM7, Bionet, Korea). PCS was performed using Perfusor Space (Perfusor^®^ space syringe pump system, B. Braun Medical Inc., Melsungen, Germany). Additionally, we recorded the bispectral index (BIS) value using Brain Monitor (BIS^™^, Covidien, USA).

Two kinds of sedative agents, propofol and midazolam, were intravenously administered at three different doses: high, middle, and low dose. After the exclusion of the low-dose group, participants were divided in total into four groups according to the two sedatives and two doses. Participants in the middle-dose groups were infused with 0.01 mg/kg midazolam or 0.3 mg/kg propofol using a lockout interval of one minute; 0.02 mg/kg midazolam or 0.5 mg/kg propofol were infused in high-dose groups using a lockout interval of three minutes. All high and middle-dose agents were infused at a rate of 1500 ml/h. The lockout interval is the period during which a subsequent dose injection is not administered to avoid over-doses.

All participants rested in the supine position on a dental chair. The procedure was performed with closed eyes. Headsets were provided to the participants for applying auditory stimuli, *i*. *e*., “press the button” with a subsequent beep sound, which was repeatedly applied at 10 s inter-stimulus intervals (ISI) on average throughout the experiment. ISI was randomly assigned within 9.0-11.0 s (step size 0.1 s) to avoid the expectation of the timing of the commands.

We set the time of loss of consciousness (LOC) as the first time when a participant did not press the button for five consecutive times. The time of recovery of consciousness (ROC) was defined as the first time when a participant pressed the button after being unconscious. Since the button press indicates that participants can hear, understand the instructions, and perform the motor task, we set the first motor response following an auditory stimulus as ROC, even though it is a semiconscious level.

**Fig 1:**
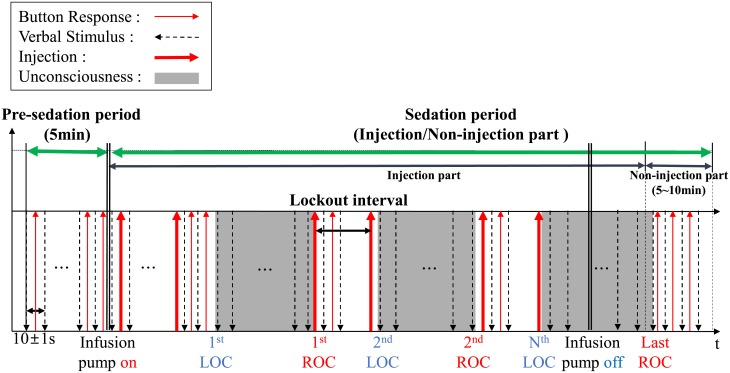
Experimental paradigm. The complete experimental paradigm with injections and different sedation levels. The designated lockout intervals for high and middle-dose groups were three minutes and one minute, respectively.

The original experiment involved a dental procedure (*i*. *e*., dental-scaling) after the above-stated sedation period (injection/non-injection part) in Fig 1. During the dental procedure phase, the subjects had their teeth scaled by a dentist to remove dental calculus using an ultrasonic scaler. To avoid the effects of noise, we excluded all dental procedure phases and analyzed only non-dental procedure phases.

### Experimental paradigm

The experiment was broadly divided into two periods: pre-sedation and sedation. During pre-sedation, the sedative agents were not injected even though participants pressed the button. The sedation period was divided into two parts: injection part and non-injection part. During the injection part (the first part), sedative agents were injected when participants pressed the button in response to a command to press the button. In this study, bolus injection with sedatives (propofol or midazolam) was performed only when the participant pressed the button without constant infusion. This method is called patient-controlled sedation [34]. This sedation technique is effective and safe when the appropriate bolus dose and lockout time is selected, because the patient will not fall into general anesthesia enough to cause side effects such as apnea, hypoxia. And, the infusion pump stopped working when a participant went into the last deep sedation level, thus the last ROC occurred without injection. The non-injection part (the second part), was the period after the non-injected last ROC where the process of commands and responses (pressing the button) lasted for more than five minutes without any injection. This whole experimental process is illustrated in Fig 1.

### Data analysis

#### Data acquisition and preprocessing

We used MATLAB (MathWorks, Natick, MA, USA) with the BBCI toolbox (http://bbci.de/toolbox), MVGC toolbox, HRVAS for data analysis [35, 36]. Using the modified International 10-20 system, EEG signals were recorded from 62 channels (FP1, FP2, AF1-4, Fz, F1-4, F7, F8, FC1-6, FT7-10, Cz, C1-6, T7, T8, CPz, CP1-6, TP7-10, Pz, P1-8, POz, PO3, PO4, PO7-10, Oz, O1, and O2), with a 1000 Hz sampling rates. The EEG reference electrode was located at FCz and the ground electrode was located at AFz. The impedance of all electrodes was kept under 10 kΩ during the experiment. The Ag/AgCl sensors were mounted on a cap (actiCAP, Brain Products GmbH, Germany). All EEG data were low-pass filtered at 40 Hz with a Chebyshev filter then downsampled to 100 Hz. We used F1 and F2, which are two frontal channels corresponding to the medial prefrontal cortex area and part of the CAN [2] commonly used for the analysis of consciousness levels [29].

ECG signals were recorded from three ECG leads with 74 Hz sampling rates (BM7, Bionet, Korea). ECG leads were fixed to the location with temporal adhesives and connected to the standard patient monitoring system. After the ECG recording, band-pass filtering was performed at 5-30 Hz to reduce noise caused by general electrical artifacts, movement artifacts and physical dynamics (*e*. *g*., electronic devices, head movement, respiration etc.). From this noise-filtered ECG, the peaks of R-waves on the ECG were detected [37]. After R-peak detection, we used cubic spline interpolation methods to convert the non-equidistantly sampled R-peak to R-peak interval (RRI) series to a new RRI series, and then, we computed the instantaneous HR with sampling rate of 50 Hz from the RRI series and extracted the mean HR from the instantaneous HR using low-pass filters at 0.04 Hz [38].

#### Comparison between the time-varying trend of the most reactive spectral band and mean HR

For time-varying trend analysis along the continuum of sedation levels, we extracted each EEG spectral power from the multitaper spectrogram to determine the change of EEG spectral power more precisely and identified a constant changing trend of the most reactive spectral band (MRSB). We verified that the results of the EEG time-frequency analysis over the different sedation levels corresponded with the previously reported results by *Purdon et al*. [29], which focused on finding EEG signatures to track consciousness levels during general anesthesia. We computed multitaper spectrograms to identify EEG dynamics in the time-frequency domain across the different sedation levels from pre-sedation (wakefulness) to deep sedation (unconsciousness). The multitaper parameters were set as the same parameters proposed by *Purdon et al*. [29]. Additionally, we compared the ratio of spectral power from each sedation level to find the MRSB, which shows the most discriminant changes among the sedation levels. From all participants, the power of each four-spectral band was averaged in all sedation levels such as pre-sedation, conscious sedation, and deep sedation. The four spectral bands consisted of theta (4-8 Hz), alpha (8-12 Hz), sigma (11-16 Hz), and beta (16-30 Hz) bands. We calculated and compared the ratio among the averaged spectral powers in all sedation levels. Based on these values, we identified more dynamically sigma power changes between pre-sedation and deep sedation than theta power changes in almost all participants. Using this comparison among all spectral bands, we determined the MRSB.

The power of the identified MRSB was extracted from the multitaper spectrogram. The MRSB power and mean HR of representative participants were compared along the continuum of sedation levels. Additionally, the smoothed MRSB power and mean HR were averaged across all participants and for comparison between those two signals. The smoothed mean HR was averaged using moving average with a window size of 100 s.

#### Granger causality analysis

To quantify the correlation between the brain and the heart, we adopted GC as a measurement for brain and heart coupling. For Granger causality (GC) connectivity analysis, we extract power spectral density (PSD) of EEG using short-time Fourier transform (STFT) [39] (the Fourier transform separately on each segment, segment length: 2 s with 1.98 s overlaps) [40]. Based on two stationary time series data, GC was computed based on predictability and precedence [41]. Conceptually, GC utilizes a linear prediction model, which was mathematically described by univariate and bivariate autoregressive models. When time series data *X* is predictable for future *X*, GC represents the degree of how much more predictable it is when another time series data *Y* is used together with *X* as prediction variates. More specifically, this can be explained by a comparison of restricted (univariate) and unrestricted (bivariate) models.

In the unrestricted model,

$$\begin{array}{c} {}[\begin{array}{c} X(t)\\ Y(t) \end{array}]=\sum_{k=1}^{p}A_{k}\cdot [\begin{array}{c} X(t-kt_{0})\\ Y(t-kt_{0}) \end{array}]+[\begin{array}{c} E_{x}\left(t\right)\\ E_{y}\left(t\right) \end{array}], \end{array}$$ (1)

where *A*_*k*_ is a bivariate 2 by 2 AR model coefficients matrix with the same *p* model order. *E*_*x*_ and *E*_*y*_ are the prediction errors generated by the bivariate AR model of *X* and *Y*. *t*_0_ is the sampling interval, which is the inverse of the sampling rate.

In the restricted model,

$$\begin{array}{c} X\left(t\right)=\sum_{k=1}^{p}B_{k}\cdot X\left(t-kt_{0}\right)+{\hat{E}}_{x}\left(t\right), \end{array}$$ (2)

where *B*_*k*_ is a univariate 1 by 1 AR model coefficients matrix with the *p* model order. $\hat{E_{x}}$ is the prediction error generated by the univariate AR model of *X*. GC values from *Y* to *X* can be explained by the log ratio of the error of predicted future *X* which is generated by univariate linear regression model of *X* to that by bivariate linear regression model of *X* and *Y*. GC values are depicted as

$$\begin{array}{c} GC_{Y\to X}=\ln[\begin{array}{c} \frac{var(\hat{E_{x}})}{var(E_{x})} \end{array}] \end{array}$$ (3)

In this study, as mentioned above, GC values were estimated between the averaged sigma power over the EEG electrodes (F1 and F2) and mean HR. GC analysis was performed on each segmented pair of the power of MRSB and mean HR along the continuum of sedation levels. To remove linear trends of data and avoid non-stationarity [42], GC analysis was applied to each pair of segmented window after detrending. And, several previous studies showed reasonable results with small sample size in GC analysis [43–45]. The length of each window was 1 s and the step size 0.5 s. Since the sampling rate of two time series data is 50 Hz, 1 s time window for GC calculation has 50 data points. From each 1 s window, using appropriate model order *p*, one GC value is obtained. GC values in (3) are generated using the variance of errors from unrestricted model (1) and from restricted model (2). This variance of error in a time window is obtained using 46∼50 error pairs, which is reasonable number to calculate the variance of error, considering GC value between other physiological signals [46]. Sedation levels were divided into four periods including wakefulness (pre-sedation without any injection), conscious sedation (from the first injection until first LOC), deep sedation (unconsciousness), and non-injection part (after non-injected last ROC). Averaged GC values of all windows involved in each period were used as the representative GC values of different sedation levels. GC values of deep sedation were the averaged GC values of all deep sedation cycles.

As stated above, the model order (*p*) appropriate for these data turned out to be four, which was determined by computing the Akaike information criterion (AIC) [47] and the Bayesian information criterion (BIC) [48] in almost all windows. In each window, the two criterions reached a minimum at the same model order. The model order was further computed for each window of each patient. The obtained model order accounts for 65% in all the calculated model orders in all windows from all participants. This GC estimation was conducted using the MVGC toolbox [35].

## Results

### EEG spectral power and mean HR

#### Time-varying power in EEG spectral bands using multitaper spectrogram

We conducted a time-frequency analysis of EEG signals of all participants to reveal trends or differences that are induced by propofol and midazolam using a multitaper spectrogram. For clear visualization of EEG spectral dynamics along the continuum of sedation levels, we extracted the time course of power in four frequency bands (theta, alpha, sigma, and beta) and observed the common patterns of PSD along the continuum of sedation levels.

**Fig 2:**
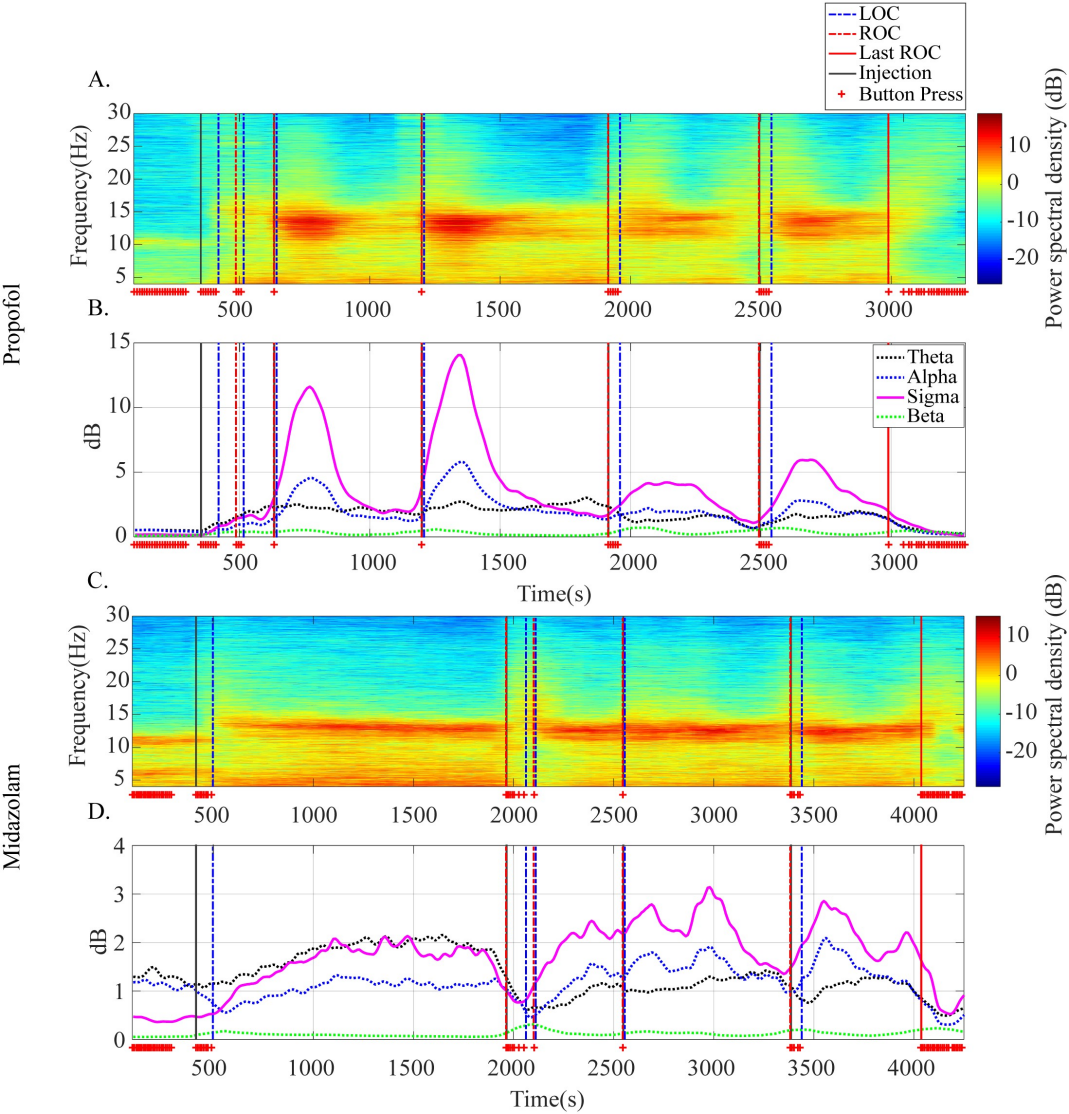
Representative time-varying electroencephalogram (EEG) spectral power along the continuum of sedation levels. EEG multitaper spectrogram (A and C) and time-varying power in four EEG frequency bands (B and D) of subject P7 in the propofol group and of participant P1 in the midazolam group. The crosses indicate a button press and a state of consciousness.

The EEG multitaper spectrogram showed clear differences in specific EEG frequency bands (10-15 Hz) along the continuum of sedation levels (Fig 2A and 2C). A clearer time course of power in each spectral band is illustrated in Fig 2B and 2D. The sigma power (11-16 Hz) showed the most sensitive changing pattern to the sedation levels by visual inspection. Sigma power increased when patients were administered the sedative agent and started to decrease at some point in deep sedation, which corresponded to the results of the multitaper spectral analysis. Sigma power decreased until the injected middle ROC and started to increase with the following injection points. Midazolam showed a similar trend, but with more fluctuating changes. We identified in representative participants that the most corresponding spectral band to the sedation level is the sigma spectral band for propofol and midazolam in Fig 2.

**Fig 3:**
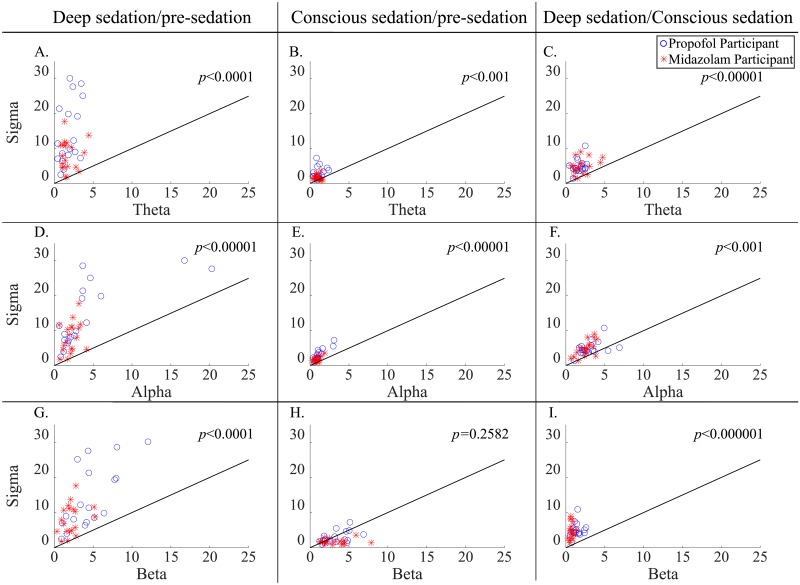
Comparison of changing ratios of the spectral power in the different sedation levels. *p*-value in each subplot indicates significant differences between each ratio of the EEG spectral power (*i*. *e*., theta, alpha, beta, and sigma) in the different sedation levels (*i*. *e*., deep sedation/pre-sedation, conscious sedation/pre-sedation, and deep sedation/conscious sedation). The diagonal line is y = x in each plot.

Fig 3 shows the MRSB, which is the most clearly changing EEG spectral band over the different sedation levels. Sigma power had the largest variation over the different sedation levels among the four frequency bands. Although the sigma power variation between pre-sedation and conscious sedation was not noticeably larger than beta power variation in Fig 3H (*p* = 0.2582), sigma power variations over the sedation levels were larger than any other spectral band variations over the sedation levels (Fig 3A, 3D and 3G for the comparison between deep sedation and pre-sedation (*p* < 0.0001); Fig 3C, 3F and 3I for the comparison between deep sedation and conscious sedation (*p* < 0.001); Fig 3B and 3E for the comparison between conscious sedation and pre-sedation (*p* < 0.001)).

#### Time-varying mean HR

**Fig 4:**
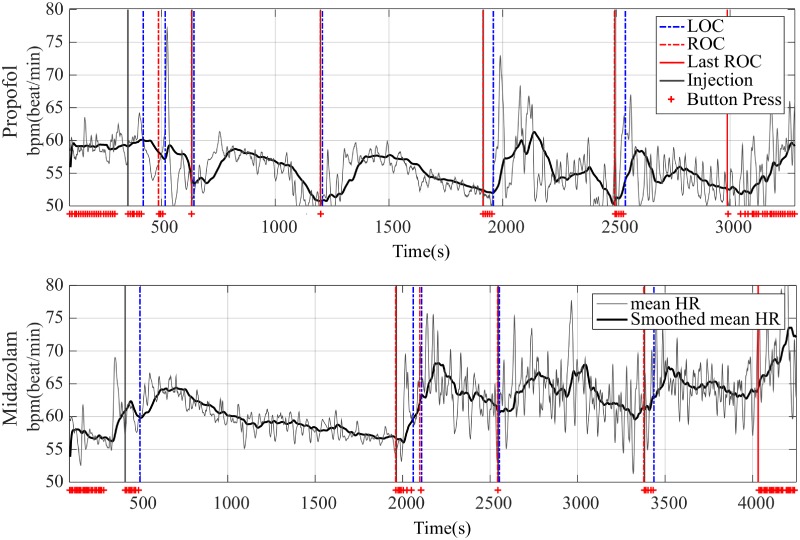
Representative mean heart rate (HR) and smoothed mean HR. Thin line for time-varying mean HR extracted using a low-pass filter at 0.04 Hz on instantaneous HR and thick line for time-varying mean HR smoothed with moving-window of participant P7 in the propofol group (top panels) and of participant P1 in the midazolam group (bottom panels).

**Fig 5:**
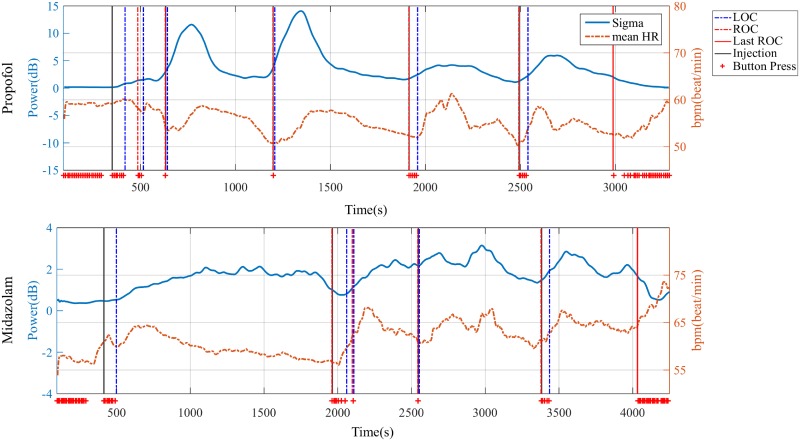
Representative overlap of sigma power and mean heart rate (HR). Comparison between sigma power and mean HR along the continuum of sedation levels of participant P7 in propofol (upper panel) and of participant P1 in midazolam (bottom panel).

Fig 4 displays the mean HR by filtering and smoothed mean HR in both propofol and midazolam groups. Similar to the EEG sigma power, the mean HR increased after LOC and started to decrease at some point in deep sedation. Decreasing mean HR started to increase at the next LOC points. From the time-varying patterns of sigma power and mean HR, we identified a correlation between the time course of these signals. Fig 5 shows that the time course of sigma power and mean HR have a correlation pattern after the first LOC and until the non-injected last ROC.

#### Grand-averaged EEG and mean HR

**Fig 6:**
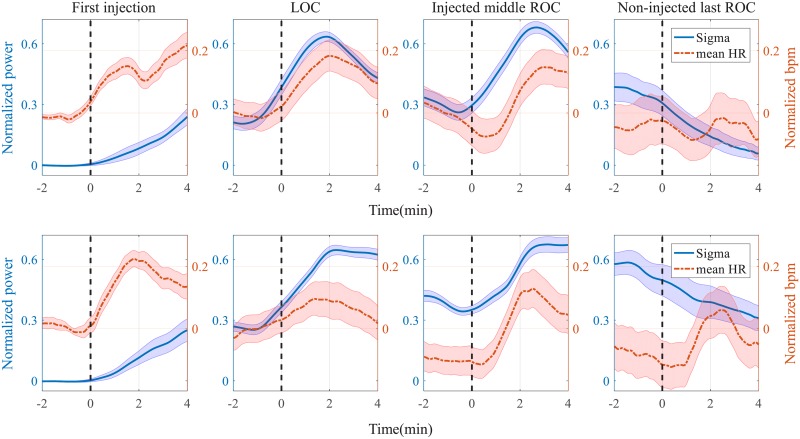
Grand-averaged sigma power and mean heart rate (HR) in particular sections (i.e., first injection, LOC, injected middle ROC, and non-injected last ROC). Comparison between sigma power and mean HR around specific points induced by propofol (top panels) and midazolam (bottom panels). The zero time-point in each subplot indicates the point of the first injection, LOC, injected middle ROC and non-injected last ROC (top line in each column of segments 1-4 respectively). The standard error of the mean is represented by the shade of the two signals.

We investigated the relationship between sigma power and mean HR in specific events on the continuum of sedation levels. Fig 6 shows the different correlation patterns between grouped sigma power and grouped mean HR over the different sedation events. In order to identify grand-averaged sigma power and mean HR patterns across the subjects, individual sigma powers and mean HRs of the whole process were normalized with minimum-maximum bound [-1 1] after baseline correction by subtracting the mean spectral power of the resting session from the whole experiment, through which normalized sigma power and mean HR in the pre-sedation period were corrected to zero-mean states.

For visualization of the grand-averaged changing trend of each signal, we extracted some segments with the same time length (six minutes), from each participant. A total of four segments were extracted from specific event points consisting of the first injection points, LOC points, injected middle ROC points, and non-injected last ROC points. All extracted segments were from -2 min to 4 min with respect to each event point and were six minutes long. Furthermore, the first injection segment and last ROC segment from all participants were grand-averaged. However, since normally three to four LOC or ROC points per participant were extracted, the LOC or middle ROC segments were first averaged in each participant. Subsequently, all the averaged LOC or middle ROC segments of one participant were once more averaged, resulting in grand-averaged LOC or middle ROC.

In the propofol group, we noted that the grand-averaged sigma power clearly started to increase at the first propofol injection point (Fig 6). After the first injection, the mean HR started to increase once again with the second propofol injection. In the grand-averaged LOC segment, sigma power and mean HR showed decreasing trends until the LOC point and showed increasing trends after the LOC point. Following the LOC segment, the sigma power and mean HR showed a similar trend in which two decreasing signals in deep sedation before ROC point reversed to increasing trends at the point of ROC in the grand-averaged ROC segment. In the non-injected last ROC segment, sigma power continuously decreased at the ROC point. In contrast, the mean HR did not show a decreasing trend, which suggests decorrelation between sigma power and mean HR.

In the midazolam group, grand-averaged sigma power also showed an increasing trend at the first injection point similar to the trend in the propofol group (Fig 6). The mean HR induced by midazolam increased, but this increasing trend was not consistent. Although sigma power and mean HR continuously increased in the LOC segment, the changes were more obvious in the sigma power compared with mean HR. In the ROC range, after the ROC injection points, both signals increased, but not in the same increasing trend. Specifically, at approximately two minutes, mean HR started to decrease, but not the sigma power. In the range of non-injected last ROC, sigma power slightly decreased as in the propofol group, but the mean HR soared at the point of non-injected last ROC and decreased at approximately three minutes.

We observed a noticeable correlation between sigma power and mean HR in both propofol and midazolam groups by visual inspection. In particular, when propofol was used, there was a clear correlation between the sigma power and mean HR along the continuum of sedation levels.

### GC analysis in different sedation levels

**Fig 7:**
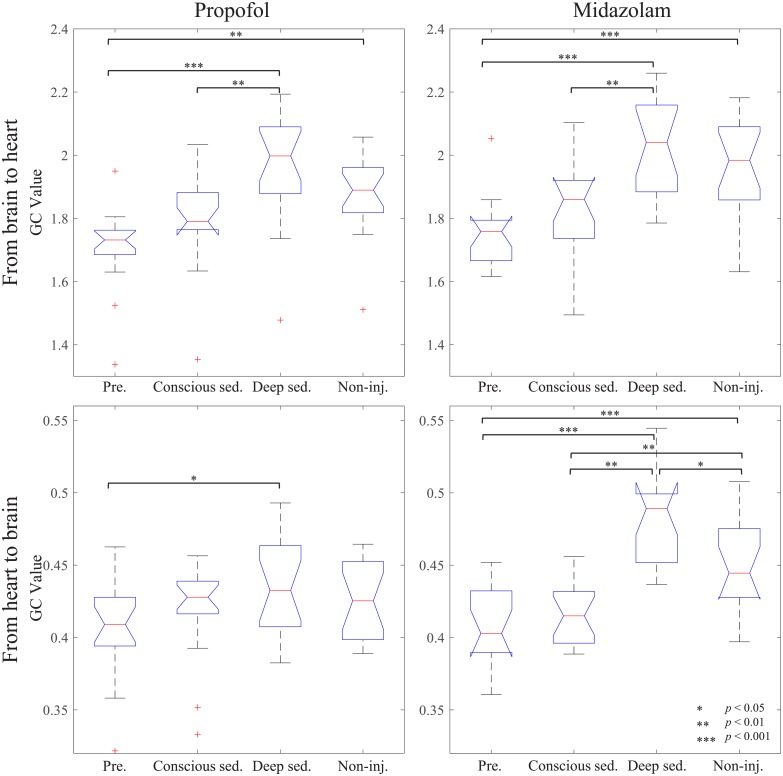
Averaged GC values at different sedation levels. Left panel for averaged GC values in the propofol group and right panel in the midazolam group. Upper panels show GC_b→h_ and bottom panels show GC_h→b_; *Pre*: pre-sedation part with wakefulness, *Conscious sed*: conscious sedation, *Deep sed*: deep sedation, and *Non-inj*: non-injection part.

Fig 7 shows the grand-averaged GC values across the subjects from the brain to the heart (GC_h→b_) GC values from the heart to the brain (GC_b→h_) in each sedation level (Table 2). We tested the normality of the data by Shapiro-Wilk test. An increase in GC_b→h_ is clearly seen in both groups of sedative agents, as the participants reached deep sedation. In the propofol group, one-way analysis of variance (ANOVA) revealed significant differences in GC_b→h_ of different sedation levels (F(3,68) = 10.45, *p* < 0.00001). In the same manner, one-way ANOVA of midazolam group also showed significant differences in the GC_b→h_ of different sedation levels (F(3,64) = 11.31, *p* < 0.00001). Paired t-test post hoc analyses followed by Tukey’s multiple comparisons [49] showed the degree of difference between each pair of GC_b→h_ in the different sedation levels, as indicated by asterisks in Fig 7. One-way ANOVA was also performed to examine the difference of GC_h→b_ among different sedation levels in both propofol (F(3,68) = 2.58, *p* = 0.0603) and midazolam (F(3,64) = 25.10, *p* < 0.00001) group. Tukey-corrected paired t-tests showed significant differences in GC_h→b_ among sedation levels. However, the GC_b→h_ and GC_h→b_ were comparable between pre-sedation and conscious sedation in both sedative agent groups, and distinguishable GC_h→b_ between conscious sedation and Non-injection in only midazolam. Similar with the GC_h→b_ case, significant difference in GC_b→h_ was also observed with the increasing trend of mean GC_b→h_ as the sedation level deepened.

**Table 2:** Mean and standard deviation (SD) of the grand-averaged GC values.

	GC_b→h_ (Mean ± SD)		GC_h→b_ (Mean ± SD)	
	Propofol	Midazolam	Propofol	Midazolam
Pre-sedation	1.71±0.13	1.75±0.11	0.41±0.03	0.40±0.03
Conscious sedation	1.80±0.16	1.83±0.16	0.42±0.03	0.42±0.02
Deep sedation	1.97±0.18	2.02±0.15	0.44±0.03	0.48±0.03
Non-injection part	1.88±0.12	1.96±0.17	0.43±0.03	0.45±0.03

## Discussion and conclusion

In this study, we identified the coupling between the brain and the heart over different sedation levels based on CAN. We showed that EEG sigma power is the most reactive EEG spectral band to sedation levels and used it together with the mean HR as the signals that represent brain and heart activities, respectively. To identify the coupling pattern between the brain and the heart along the continuum of sedation levels, we induced dynamically changing sedation levels using PCS with two different sedative agents, propofol and midazolam. Based on our results, the sigma power showed noticeable time-varying correlation with the mean HR. The coupling pattern differed significantly among the different sedation levels as assessed by GC value.

First, we revealed that the sigma band is the optimal and representative frequency band to reflect the change in the sedation levels and the specific experimental protocol studied here. The individual participants in each sedative group in Fig 2 show that the sigma band power is the obviously changing band over the continuum of sedation levels; this is also demonstrated as a common feature of all participants in Fig 3. The reactivity of the sigma band can also be seen in the sleep spindle. The sigma band involving sleep spindles is commonly known to be generated by the thalamus and are known to spread through the thalamocortical network [50–52]. Propofol and midazolam are known to induce sedation by affecting the GABA receptors in the thalamus and thalamocortical network [53–55]. Therefore, we assume that the thalamus was affected by the two sedatives and contributed to the generation of sigma spectral power.

Second, we verified the coupling pattern between the brain and the heart along the continuum of sedation levels, showing a time-varying correlation between sigma power and mean HR. PCS generated dynamically changing sedation levels and sigma power demonstrated this consciousness dynamics by repeated waxing and waning with the LOC, ROC, and injection points. The mean HR followed the dynamics of sigma power along the sedation levels. Figs 5 and 6 show these individual and group-averaged correlation trends, respectively. However, this time-varying correlation was interrupted in the non-injected last ROC, *i*. *e*. non-injection part. Based on our results, there are some differences in the correlation pattern between the two sedative agents.

In the midazolam group, the overall trend of both signals did not show a clear correlation like in the propofol group. These sigma power and mean HR trends of the midazolam group were generally identical with the respective trends of the propofol group. However, a more detailed analysis demonstrated that the sigma power and mean HR in the midazolam case show an unclearer trend of increase and decrease along the continuum of sedation levels than the trends of the sigma power and mean HR in the propofol group. The sigma power and mean HR in propofol group show a repetitive increase-and-decrease trend along the continuum of the sedation levels. However, sigma power and mean HR in the midazolam group do not show this clear increasing and decreasing trend. Nevertheless, the grand-averaged mean HR in the midazolam group also showed a similar trend with sigma power except for the non-injected last ROC segment. We attribute this difference between the propofol and midazolam groups to the more pronounced changes in EEG and mean HR in the midazolam group compared with the propofol group. Individual trends (Fig 2D and bottom panel of Fig 4) showed that many of the midazolam group participants had fluctuating EEG and mean HR, even though the sigma power of all participants in all groups was extracted using the same spectrogram methods and smoothing filter for visualization of mean HR, which offsets increasing or decreasing trends in the grand-average process (Fig 6).

Despite the difference between propofol and midazolam, the correlation patterns between sigma and mean HR are in line with previous studies about the association between the cortex and autonomic processing [56–58]. For example, previous reports using time-series fMRI [59, 60] demonstrated that the connectivity within frontal cortical areas or the ventral medial prefrontal cortex is strongly correlated with HR dynamics, which suggests that the ventral medial prefrontal cortex is involved in controlling HR. Based on this, we hypothesize that there may be a connectivity or route for information flow between the brain and the heart. Even though this interaction between the brain and heart was demonstrated on the basis of resting states or motor task states, it could be possible to support our results of time-varying correlation between prefrontal sigma power and mean HR. Based on previous studies suggesting the importance of the prefrontal cortex as a modulator of autonomic processing, we observed that the sigma power of prefrontal areas, which repeatedly change owing to the effects of sedatives, affect mean HR, which can be a gateway between subcortical regions and the sinoatrial node of the heart [61, 62].

Most previous studies reporting on brain-heart connectivity have assumed the route of physiological information flow using signals presenting the organs’ activities [5, 13, 14]. In addition, it is possible to infer a network change between the brain and other organs depending on the level of consciousness in several studies [14, 32]. We observed that our results of the GC values according to the level of sedation, which corresponded to the results of previous studies. However, we cannot demonstrate the inner pathway where physiological information between brain and heart flows. We can only show interaction through the superficial coupling between the final output signals of brain and heart activities but cannot verify inner pathways linking brain and heart. Although we cannot figure out physiological inner pathway between the brain and heart generating EEG and ECG signals, we tried to show the interaction flow indirectly through time-varying coupling analysis between EEG and ECG signals according to the depth of consciousness. Our results show the different degree of interaction between cortex and heart activity according to different sedation levels. Those results sufficiently meet our anticipation. However, this is difficult to reveal direct and clear functional interaction between frontal cortex and heart. The GC value between the EEG and heart activity can show a correlation between the signals of the frontal cortex and heart, which requires cautiousness for interpretation.

The time-varying correlation was further analyzed using GC to explore differences of correlation among the various sedation levels and directions of information flow. GC-based coupling estimation was used as a measure of causality level and gave more insights about the directional influence between the brain and the heart. From this GC-based coupling analysis, we expected to find directional information flow between the brain and the heart. Therefore, we focused on the difference in GC values among the different sedation levels. In both the propofol and midazolam groups, the mean of GC_b→h_ increased as the depth of sedation level deepened. And, GC_h→b_ were much lower than GC_b→h_ in all sedation levels. Based on these results, we observed that the time-varying causal link pattern between the sigma power and mean HR was clearly different between the different sedation levels and between the directions of information flow.

Meanwhile, the GC is widely used methodology in neuroscience to discern connection among other areas of brain. GC in neuroscience field is mainly used for analysis of fMRI image data and EEG data. However, the GC should be analyzed with great caution because of an entailed list of the controversial issues. In particular, measurement noise can reverse the estimation of causality direction and the temporal smoothing can induce spurious causality [63]. EEG and ECG used in our study are known to be susceptible to noise due to their properties. In particular, the EEG is sensitive to external noise (*e*. *g*., head and eye movement, etc.) as a micro signals of less than 50 *μ*V. This noise usually affects slow waves (< 2 Hz), which does not effect on the sigma band of brain signal. In addition, the mean HR was used for GC estimation after converting the ECG signal to HR which is relatively unaffected by noise. In short, our obtained GC values can show the interaction between frontal cortex and heart using sigma power and mean HR, which are less noisy. Therefore, even though GC may be vulnerable to noise, our GC value is likely to be used as proper coupling factor.

In our GC analysis results, the non-injection part GC_b→h_ was significantly higher than the pre-sedation part in both the propofol and midazolam groups. However, the participant responds to verbal stimuli in these both parts (the pre-sedation and non-injection part). We can assume that the reason for this phenomenon was the presence of sedative residues, considering the non-significant differences between the GC_b→h_ value in the conscious sedation and non-injection part. The conscious sedation and non-injection parts involve mild or moderate sedation owing to the residues of sedative agents, which made it difficult to distinguish the non-injection part from consciousness and deep sedation. Conversely, this made it easy to distinguish the non-injection part from the pre-sedation part. We can assume that the sedative is an essential component for determining GC_b→h_ value.

In contrast, the mean GC_h→b_ in deep sedation was the highest among all other sedation levels in both sedative agent groups (Table 2). In addition, GC_h→b_ can significantly discriminate deep sedation from the pre-sedation part, similar with the GC_b→h_. In particular, we can also observe noticeably different GC_h→b_ value with respect to the different sedation levels in midazolam group. We can assume that the information flow from the heart to the brain might be shown in GC_h→b_ analysis since the thalamus also communicates the visceral information to the insula and medial prefrontal areas [64]. These results are in agreement with those of previous studies about visceral responses in cortical sensory areas during sleep [65–70]. In these studies, it was revealed that some cortical areas responded to visceral activities including heart activity during sleep, suggesting that visceral projections to some cortical areas are activated only during sleep [65–70]. Visceral projections to cortical areas can be an evidence of directional information flow from the heart to the brain. Additionally, much stronger visceral projections to the cortical areas during sleep than during awake states are in accordance with our results where mean GC_h→b_ was higher in deep sedation than the other consciousness states. In particular, GC_h→b_ values in deep sedation which means unconsciousness state show significant differences with pre-sedation levels, regardless of the sedative agents. From this, we assume that our results might be affected by visceral projections.

GC analysis provided directional information between the two organs as a concept of the effective connectivity and demonstrated that the coupling between sigma power and mean HR becomes stronger with deeper sedation levels. In particular, based on the significantly higher GC_b→h_ values than GC_h→b_ values at all sedation levels, we can assume that the time-varying causal link between sigma power and mean HR is more attributed to sigma power than mean HR. This result of GC analysis can be interpreted that there are causal links between the sigma power and mean HR and this strength of causal link is different for each direction of link and sedation level.

In present study, we investigated the coupling between spectral power and mean HR from the cortical and cardiac activities, respectively. Even though the signals were extracted from two prefrontal EEG electrodes and three ECG leads, which is typical anesthetic operation setting for safety, the discriminable coupling strength between brain and heart presents the potential for a more practical and efficient application to monitor the depth of sedation in the clinical practice. However, the proposed method is not able to guarantee the reliability in the facial surgery (*e*. *g*., plastic surgery, etc.). In future studies, we should investigate the handling of noise issue for developing a universal depth of sedation monitoring system.
